# [^18^F]Fluorocholine PET/MRI and [^18^F]Fluorocholine PET/CT as first- and second-line imaging in primary hyperparathyroidism – who takes the lead?

**DOI:** 10.1007/s00259-025-07373-1

**Published:** 2025-05-30

**Authors:** Petra Petranović Ovčariček, Jan Schaab, Stephan Beintner-Skawran, Alexander Maurer, Niels J. Rupp, Kristian Ikenberg, Grégoire B. Morand, Simon A. Mueller, Diana Vetter, Virginia Liberini, Luca Giovanella, Martin W. Huellner

**Affiliations:** 1 Department of Oncology and Nuclear Medicine, University Hospital Center Sestre Milosrdnice, Zagreb, Croatia; 2 School of Medicine, University of Zagreb, Zagreb, Croatia; 3 Department of Nuclear Medicine, University Hospital Zurich, University of Zurich, Zurich, Switzerland; 4 Department of Pathology and Molecular Pathology, University Hospital Zurich, University of Zurich, Zurich, Switzerland; 5 Faculty of Medicine, University of Zurich, Zurich, Switzerland; 6 Department of Otorhinolaryngology, Head and Neck Surgery, University Hospital Zurich, University of Zurich, Zurich, Switzerland; 7 Department of Visceral and Transplant Surgery, University Hospital Zurich, University of Zurich, Zurich, Switzerland; 8 Nuclear Medicine Unit, ASO Santa Croce e Carle Cuneo, Cuneo, Italy; 9 Department of Nuclear Medicine, Gruppo Ospedaliero Moncucco SA, Clinica Moncucco, Lugano, Switzerland

**Keywords:** ^18^F-fuorocholine, PET, Computed tomography, Magnetic resonance imaging, Primary hyperparathyroidism, Parathyroid adenoma

## Abstract

**Purpose:**

Accurate preoperative localization of hyperfunctioning parathyroid glands is essential for enabling a focused surgical approach in patients with primary hyperparathyroidism (pHPT). Our study aims to determine the diagnostic value of ^18^F-fluorocholine positron emission tomography/magnetic resonance imaging ([^18^F]FCH PET/MRI) and [^18^F]FCH PET/computed tomography (PET/CT) in identifying parathyroid adenomas.

**Methods:**

We conducted a retrospective study to assess the diagnostic accuracy of [^18^F]FCH PET/MRI and [^18^F]FCH PET/CT as first- and second-line methods for detecting parathyroid adenomas in patients with pHPT. The primary endpoints included patient-based sensitivity, specificity, and positive predictive value (PPV), with intraoperative surgical localization and histopathological analysis serving as reference, i.e., gold standard.

**Results:**

The study included 322 patients. A total of 264 patients underwent [^18^F]FCH PET/MRI, thereof 155 and 109 as a second- and first-line method, respectively. [^18^F]FCH PET/CT was performed in 58 patients, thereof 44 and 14 in a second- and first-line setting, respectively. Total sensitivity, specificity, PPV, negative predictive value (NPV), and diagnostic accuracy of [^18^F]FCH PET/MRI were 0.967, 0.818, 0.983, 0.692, and 0.955, respectively, while the same parameters for [^18^F]FCH PET/CT were 0.980, 0.875, 0.980, 0.875, and 0.966, respectively (all *p* > 0.05). There was also no difference between the two methods in the first- or second-line imaging scenarios.

**Conclusion:**

Both [^18^F]FCH PET/MRI and [^18^F]FCH PET/CT are highly accurate imaging modalities for localizing parathyroid adenomas in both first- and second-line settings. They can be regarded as “top-scoring” or “one-stop-shop” techniques for preoperative assessment.

## Introduction

Primary hyperparathyroidism is a relatively common endocrine disorder, with an incidence of approximately 45 cases per 100,000 person per year [1]. It results from hyperfunctioning parathyroid gland(s) and typically presents with hypercalcemia, elevated serum parathyroid hormone (PTH) levels, and hypercalciuria, although, in rare cases, serum PTH or calcium levels may remain within the reference range [2, 3]. Hyperfunctioning parathyroids are most often solitary lesions, though multiple adenomas or parathyroid gland hyperplasia occur in up to one-fifth of cases, while parathyroid carcinoma remains exceptionally rare [4, 5]. Surgical removal of the hyperfunctioning glands is the only curative treatment [6, 7]. The success of surgery relies heavily on precise preoperative localization, which facilitates a more targeted approach [8]. A focused surgical strategy, such as minimally invasive parathyroidectomy (MIP), offers advantages, including shorter operative times, fewer perioperative complications, improved cosmetic outcomes, and reduced overall hospital costs [9–11].

Several imaging techniques are currently used to identify hyperfunctioning parathyroid glands. Most institutions still rely on conventional imaging, typically a combination of ^99 m^Tc-2-methoxyisobutylisonitrile single photon emission computed tomography/computed tomography ([^99 m^Tc]Tc-MIBI SPECT/CT) and cervical ultrasonography (US) [12]. A meta-analysis by Treglia et al., which included 23 studies with 1,236 patients with pHPT, reported a patient-based pooled detection rate of 88% for [^99 m^Tc]Tc-MIBI SPECT/(CT) [13]. However, when standard imaging yields negative or inconclusive results, additional advanced imaging is often required to localize hyperfunctioning glands precisely. Otherwise, bilateral neck exploration may be necessary, with increased operative morbidity compared to focused parathyroidectomy. [^18^F]FCH PET/CT is increasingly replacing conventional imaging modalities as a “one-stop-shop” modality due to its higher accuracy [14]. Numerous studies have demonstrated its superiority over traditional scintigraphic techniques [15–22]. Additionally, [^18^F]FCH PET can be integrated with MRI in hybrid [^18^F]FCH PET/MRI systems. This combination offers higher soft tissue contrast than PET/CT and has shown excellent accuracy compared to hybrid systems based on CT and in current smaller-scale sample studies, comparable to [^18^F]FCH PET/CT [23–25]. The superior anatomic detail provided by MRI may be particularly beneficial in preoperative planning and in cases where parathyroid glands exhibit low [^18^F]FCH uptake [26, 27]. Moreover, MRI has the advantage of no radiation exposure, making it a safer alternative for certain patient groups, such as pregnant women and younger individuals, where radiation dose considerations are critical. The primary purpose of our study was to evaluate and compare the diagnostic performance of [¹⁸F]FCH PET/MRI and [¹⁸F]FCH PET/CT in accurately detecting and localizing parathyroid adenomas in patients with pHPT. We specifically assessed these modalities in first- and second-line imaging settings to determine their efficacy and potential advantages in preoperative localization.

## Methods

### Patients

In this retrospective study, we analyzed patients with suspected pHPT who underwent [^18^F]FCH PET/CT or [^18^F]FCH PET/MRI at University Hospital Zurich from November 2013 to November 2023.

In the early years of the study, patients predominantly underwent PET/CT imaging. Over time, however, most participants were transitioned to PET/MRI due to its superior soft tissue contrast and reduced radiation exposure. Patients with contraindications to MRI or severe claustrophobia continued to receive PET/CT in later years. Additionally, PET/CT was used as an alternative when PET/MRI appointment slots were unavailable.

Patients were included if they had intraoperative surgical localization and histopathological confirmation of parathyroid adenoma as reference standards.

Patients diagnosed with secondary or tertiary HPT and those with histopathology findings of pure hyperplasia and parathyroid carcinoma were excluded.

The study was conducted in accordance with the ethical standards of the institutional Ethics Committee and the Helsinki Declaration from 1975, revised in 2013. Ethical approval was granted by the Cantonal Ethics Committee Zurich (BASEC 2022 − 01778), and only patients with signed written general consent for research purposes were included from 2016. Earlier cases were included if no documented refusal existed (ethical consent surrogate).

## [^18^F]FCH PET/CT imaging

Patients were imaged using an integrated PET/CT system (Discovery 690 Standard or Discovery MI in 5-ring or 6-ring configuration; GE HealthCare, Waukesha WI). Each patient received a standardized activity of approximately 150 MBq of [^18^F]FCH (i.e., 149.8 ± 12.2 MBq). Imaging typically started 50 min after radiopharmaceutical administration. However, in the early years of parathyroid imaging with this hybrid modality, some patients also underwent an early imaging acquisition at 2 min post-injection. The PET acquisition was performed for 3 min per bed position, covering 2–4 bed positions per patient, from the vertex to the diaphragm. However, studies demonstrated that early-phase images provide no added value, leading to a transition to late-phase imaging only in subsequent examinations. The late-phase PET acquisition followed the same acquisition parameters. All PET datasets underwent standardized corrections for dead time, random, scatter, and attenuation. Image reconstruction was performed using 3D time-of-flight, ordered subset expectation maximization (OSEM), 3 iterations and 18 subsets (matrix size of 256 × 256 pixels, axial field of view: 153 mm), or block sequential regularized expectation maximization (BSREM, β value of 300), depending on the scanner type.

A low-dose CT scan (100 mAs, 120 kV, slice thickness 3.75 mm) was acquired, as well as a diagnostic contrast-enhanced CT scan of the neck if no contraindications applied.

The total examination procedure, from initial patient positioning through the completion of image acquisition, required a maximum of 19 min.

## [^18^F]FCH PET/MR imaging

Patients were imaged using an integrated PET/MRI system (GE HealthCare, SIGNA PET/MR) equipped with a 3 T MRI and a time-of-flight PET scanner. The injected activity (approximately 150 MBq of [^18^F]FCH, i.e., 147.8 ± 13.2 MBq) and PET imaging parameters were consistent with those used in the previously mentioned PET/CT technique. For attenuation correction, an axially acquired T1-weighted Dixon-type sequence was used. The MRI protocol included the following pulse sequences: T1-weighted liver volume acquisition flexible (LAVA-flex) and coronal T2-weighted for the area from the vertex to the diaphragm), and several pulse sequences for the neck and upper mediastinum (axial T1-weighted fast spin echo, axial T2-weighted with a decomposition of fat and water and least squares estimation and echo asymmetry, coronal T2-weighted combined with short tau inversion recovery, coronal T2-weighted fast-relaxation, and fast spin echo with a saturation of fat. The selected sequences reflect a diagnostic head and neck MRI protocol, excluding contrast-enhanced, diffusion-weighted, and perfusion imaging. Sequence selection was guided by the common trade-off between acquisition time and sufficient image quality. Given that PET was the primary modality for detecting small lesions, MRI pulse sequences were chosen to optimize anatomical context and overall image quality. In this regard, 2D sequences were preferred over 3D isotropic sequences due to their typically higher in-plane resolution and faster acquisition times.

PET/MR imaging started 50 min after the radiopharmaceutical was administered. Patients were scanned from the vertex to the diaphragm. The PET images were corrected in the same way as detailed above. The PET image reconstruction was performed using 3D time-of-flight ordered subset expectation maximization with 2 iterations and 28 subsets (matrix size of 256 × 256 pixels; acquisition time, 3 min per bed position; 2 bed positions per patient; axial field of view 153 mm), and/or block sequential regularized expectation maximization (β value of 200). Although the β values used for PET/CT and PET/MRI reconstructions differ, it is important to note that these values are not directly comparable across scanner platforms. Instead, image quality should be evaluated based on the visual impression of the reconstructed images. In this context, both β values yielded comparable image quality, as determined in preliminary testing.

Contrast-enhanced images were not acquired since it was demonstrated that contract agents do not provide additional value when MRI is combined with [^18^F]FCH PET [28]. As a result, our MRI protocol utilized unenhanced anatomical T1- and T2-weighted images.

The total examination time, from initial patient positioning through the completion of image acquisition, was 32 min on average.

## Image interpretation

The [^18^F]FCH PET/CT and [^18^F]FCH PET/MRI images were evaluated by two doubly board-certified, experienced nuclear medicine physicians/radiologists in a consensus conference. The presence and localization of focal [^18^F]FCH uptake suspicious for parathyroid glands were recorded, with classifications as left, right, or ectopic relative to the thyroid. CT and MRI were used for anatomical correlation. Since the polarity of adenomas (upper/lower pole origin) often cannot be reliably determined on preoperative imaging and since the surgical approach is vastly determined by the presence/absence, sidedness and the actual anatomical level of an adenoma, and not necessarily by its polarity, our study did not take into account the polarity of adenomas, but besides presence/absence focused only on their sidedness.

## Surgery and histopathology

All but 26 patients underwent parathyroidectomy within two months of PET/CT or PET/MRI, performed by an endocrine surgeon or a dedicated head and neck surgeon. The parathyroidectomy was typically performed as a focused parathyroidectomy in cases of a single positive adenoma. For multiple or ectopic lesions, the surgical approach was adjusted based on the imaging results and intraoperative PTH levels at the surgeon’s discretion. Intraoperative PTH was measured before surgery and after the excision of the adenoma. If imaging results were negative, a conventional bilateral neck exploration was performed, or it was refrained from surgery, depending on the clinical situation. A decrease of intraoperative PTH of > 50% and a drop to within the reference range were considered appropriate [29, 30]. The exact intraoperative localization of the removed parathyroid glands was reported for each specimen, and histopathology was conducted by endocrine pathologists using hematoxylin/eosin staining of formalin-fixed and paraffin-embedded (FFPE) sections. Immunohistochemistry with anti-PTH antibodies was used if necessary. Our study included patients with parathyroid adenomas only, excluding those with parathyroid hyperplasia. The diagnosis of ‘adenoma’ versus ‘hyperplasia’ was always made by the pathologist in conjunction with clinical and radiological findings, as a definitive distinction between the two cannot be reliably established based on a single histological specimen alone. Both lie on a spectrum of hyperfunctioning parathyroid tissue - parathyroid carcinoma being at the far end of this continuum. At our institution, the vast majority of such lesions are diagnosed as “adenoma” rather than “hyperplasia,” with imaging findings taken into account during diagnosis. We acknowledge that classification practices may vary between institutions. Parathyroid carcinoma, which is exceedingly rare and more readily distinguishable due to its invasive behavior, was not included in our cohort.

### Definitions

First-line imaging refers to when either [¹⁸F]FCH PET/MRI or [¹⁸F]FCH PET/CT was used as the initial diagnostic imaging method for patients with suspected pHPT. In contrast, second-line imaging refers to when patients first underwent conventional scintigraphic imaging, but these methods yielded inconclusive or negative results, prompting subsequent second-line [¹⁸F]FCH PET/MRI or [¹⁸F]FCH PET/CT to localize parathyroid adenomas. Our study assessed the diagnostic performance of both PET modalities to determine their efficacy as first-line or second-line imaging technique.

Patient-based localization was considered true positive if all lesions on imaging matched the surgical report and were confirmed by histopathology as parathyroid adenomas. A true negative result was defined as concordant negative imaging and negative surgical findings.

A false positive result was classified in cases where imaging showed a positive finding but the surgical report was negative or where imaging and surgery showed a positive result but histology was negative. It also included situations where surgery and histopathology identified parathyroid adenomas, but the lesions were located in a different site than indicated by imaging.

A false negative was defined as negative imaging results despite positive surgical and histopathological findings. Additionally, patients who were imaging-negative and had not undergone surgery were also considered false negatives.

Ectopic parathyroid glands were defined as those not positioned in the typical locations in relation to the thyroid. Therefore, ectopic locations included all other (non-orthotopic) locations, such as inside the thymus, inside the thyroid gland or within its capsule (intrathyroidal), the tracheoesophageal groove, in the carotid sheath, behind the oesophagus, in the mediastinum (outside the thymus), in the pericardium, or undescended glands higher up in the neck.

### Data analysis

The current sample size was determined to be sufficient based on the estimated sensitivity, specificity, PPV, and NPV values of previous [^18^F]FCH studies for the detection of parathyroid adenoma, a predetermined acceptable margin of error or confidence interval width, and a level of statistical power for detecting differences between the modalities.

Descriptive statistics data analysis was performed. Quantitative data were described with mean, and standard deviation, median and interquartile range (IQR), while qualitative data were described with numbers and corresponding percentages. Sensitivity, specificity, PPV, NPV, and accuracy were calculated on a patient-based level.

McNemar test was used to compare the diagnostic accuracy of [^18^F]FCH PET/MR and [^18^F]FCH PET/CT.

Kruskal-Wallis test was performed to assess the difference in PTH level between various locations of adenomas due to the non-normal distribution of PTH values. Multiple comparisons were conducted to evaluate differences between specific adenoma location groups: (1) overall comparison across all location types (no adenoma, left, right, ectopic, bilateral, and combined orthotopic/ectopic), (2) comparison between left, right, ectopic, and bilateral locations, (3) comparison between ectopic, left, and right locations, (4) comparison between left, right, and bilateral locations, and (5) direct comparison between left and right adenoma locations.

Mann-Whitney test compared age, PTH level, and radiotracer activity among patients who underwent [^18^F]FCH PET/MR and [^18^F]FCH PET/CT. Statistical significance was set at *p* < 0.05. All statistical analyses were performed using SPSS version 26.0 (IBM Corp., Armonk, NY, USA), and post-hoc pairwise comparisons with Bonferroni correction were applied where the Kruskal-Wallis test indicated significant differences.

## Results

Our study included 322 patients, comprising 255 females (79.2%) and 67 males, with a median age of 64.8 years (IQR 72.7–55.4). The median preoperative PTH level was 105.3 ng/L (IQR 140.00–84.93).

In 199 cases, patients first underwent conventional imaging with [^99 m^Tc]Tc-MIBI SPECT/CT and cervical US, performed by institutional or external nuclear medicine physicians/radiologists. However, when these first-line imaging methods yielded inconclusive or negative results, patients were referred to [^18^F]FCH PET/CT or PET/MRI imaging as a second-line imaging approach.

A total of 264 patients underwent [^18^F]FCH PET/MRI, with 155 as a second-line and 109 as a first-line imaging method. Meanwhile, 58 patients underwent [^18^F]FCH PET/CT, with 44 as a second-line and 14 as a first-line imaging method (Table 1).

**Table 1:** Patient distribution by imaging method and setting, age, PTH level, and administered activity by method

Imaging method	total patients	first-line setting	second-line setting	Age (mean ± SD)	PTH level (mean ± SD)	MBq level (mean ± SD)
[^18^F]FCH PET/MRI	264	109	155	61.9 ± 14.3	138.1 ± 159.7	147.8 ± 13.2
[^18^F]FCH PET/CT	58	14	44	65.7 ± 14.8	223.5 ± 359.5	149.8 ± 12.2
Total/p-value	322*	123*	199*	0.020**	0.230**	0.134**

[^18^F]FCH PET/MRI,^18^F-fuorocholine positron emission tomography/magnetic resonance imaging; [^18^F]FCH PET/CT, ^18^F-fuorocholine positron emission tomography/computed tomography; PTH, parathyroid hormone; MBq, Megabecquerel; SD standard deviation; *, Total; **, *p*-value

The study showed a statistically significant age difference between patients who underwent [¹⁸F]FCH PET/MRI and those who underwent [¹⁸F]FCH PET/CT (*p* = 0.020). Patients in the PET/CT group were older, with a mean age of 65.7 ± 14.8 years, compared to patients in the PET/MRI group with a mean age of 61.9 ± 14.3 years. 

**Fig. 1:**
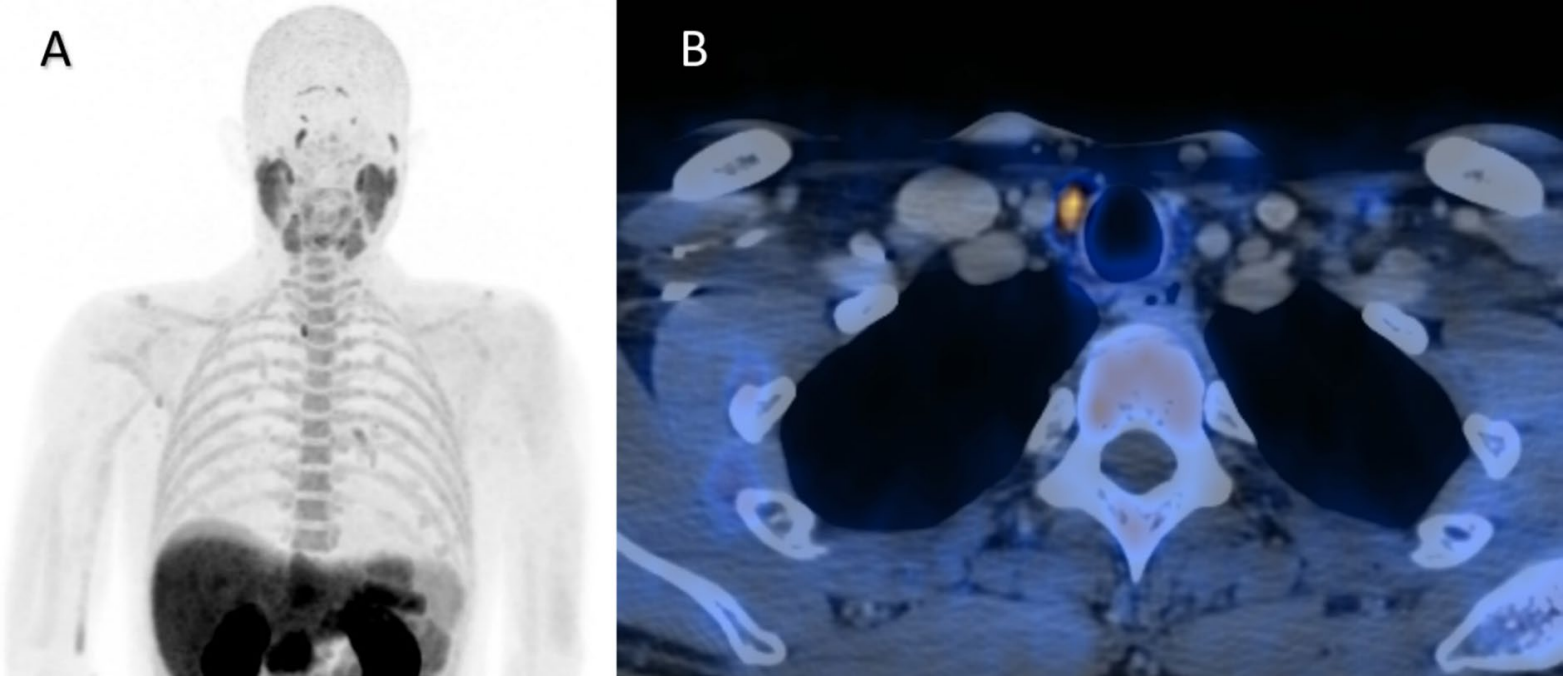
Right-sided orthotopic adenoma. Legend: 46-year-old man with pHPT, albumin-corrected serum calcium level 2.62 mmol/L (2.19–2.54 mmol/L), PTH 96.4 ng/L (15–65 ng/L). MIP (**A**) and contrast-enhanced axial PET/CT (**B**) image show a right-sided lower pole parathyroid adenoma (SUVmax 7.9, 11 × 7 × 5 mm), confirmed by surgery

**Fig. 2:**
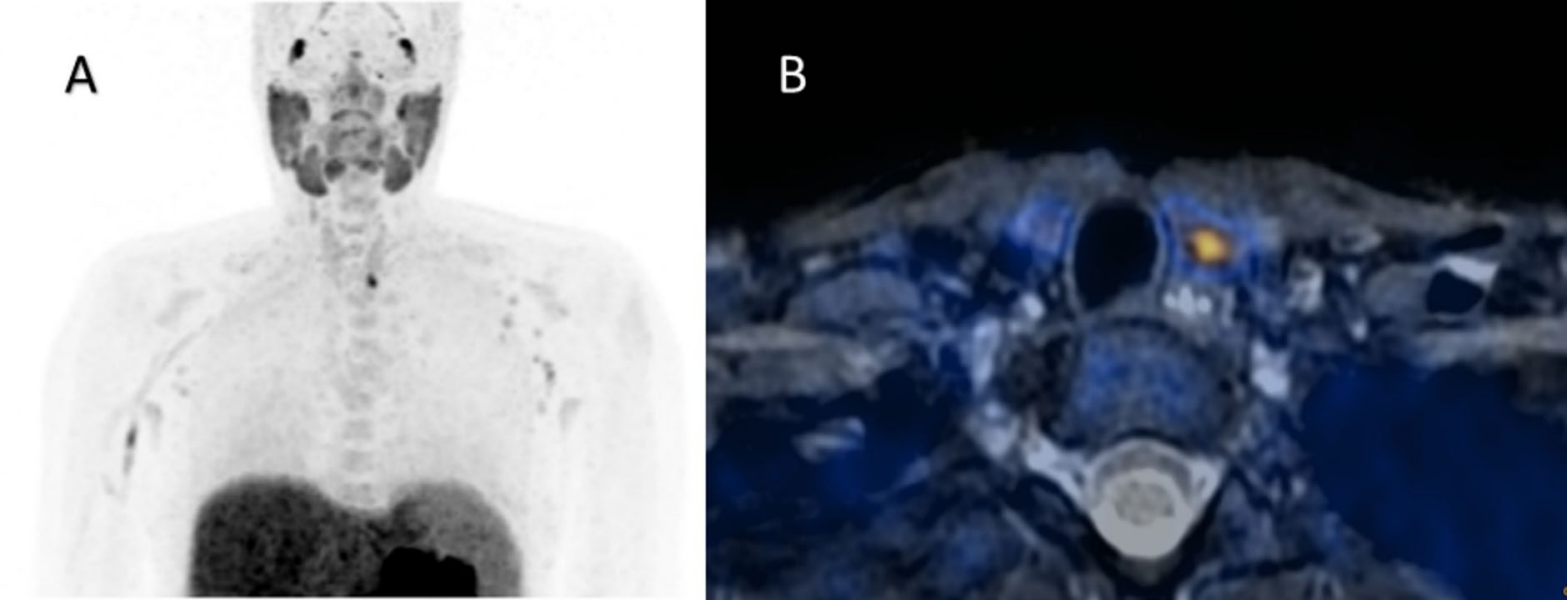
Intrathyroidal parathyroid adenoma. Legend:24-year-old woman with pHPT, albumin-corrected serum calcium level 2.64 mmol/L (2.19 – 2.54 mmol/L), PTH 107.1 ng/L (15-65 ng/L). MIP (**A**) and T2w-weighted fat-suppressed axial PET/MR (**B**) image show a left-sided intrathyroidal, intraparenchymal parathyroid adenoma (SUVmax 13.0, 6x4x6mm), confirmed by surgery

**Fig. 3:**
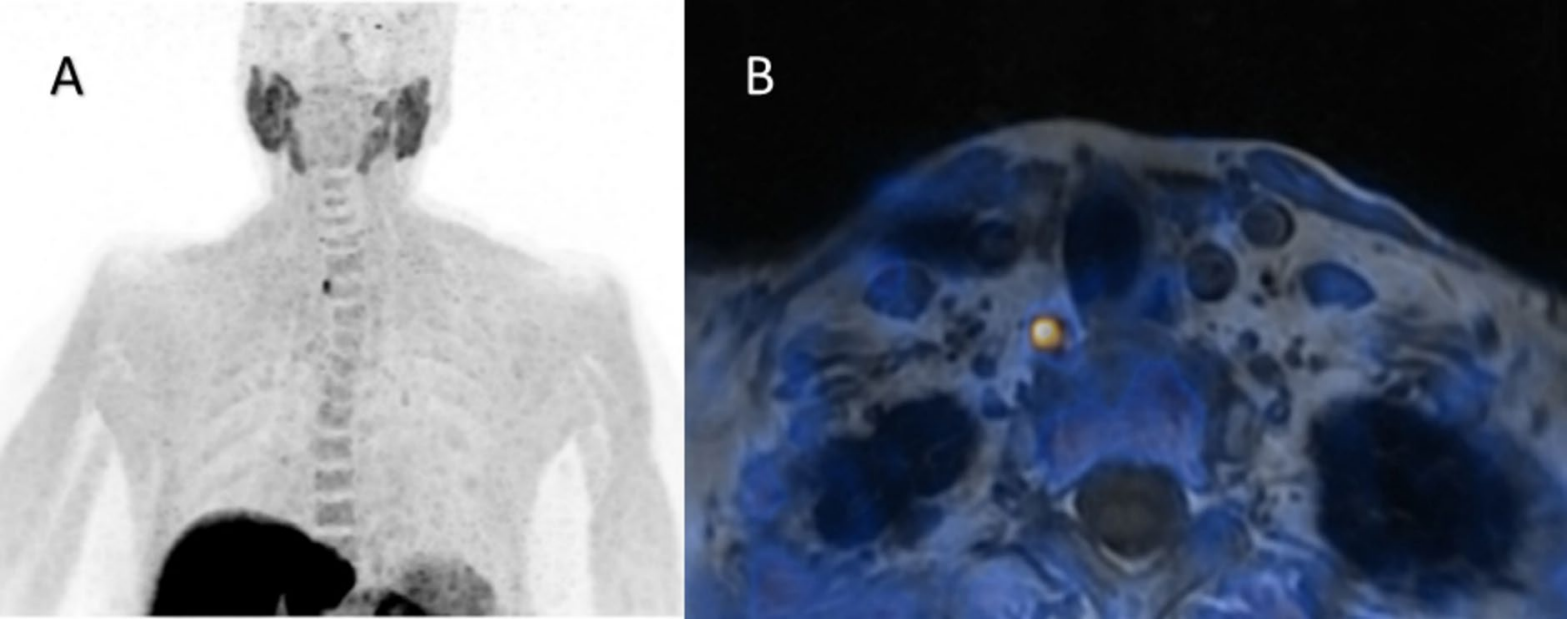
Overly descended right-sided parathyroid adenoma. Legend: 75-year-old woman with pHPT and a history of relapsing kidney stones as well as osteoporotic fractures, albumin-corrected serum calcium level 2.65 mmol/L (2.19 – 2.54 mmol/L), PTH 141.0 ng/L (15-65 ng/L). MIP (**A**) and T1w-weighted axial PET/MR (**B**) image show an overly descended right-sided upper pole parathyroid adenoma (SUVmax 10.8, 11x6x6 mm), confirmed by surgery

Out of 322 patients, 30 had no adenoma on [^18^F]FCH PET/CT or PET/MR, while 113 had left-sided orthotopic adenomas, 143 had right-sided orthotopic adenomas (Fig. 1), and 24 had bilateral orthotopic adenomas, 9 had exclusively ectopic adenomas (Figs. 2 and 3), and 3 had a combination of unilateral orthotopic and ectopic adenomas.

The corresponding PTH values were 94.0 +/- 73.4 ng/L,125.9 +/- 86.6 ng/L,141.8 +/- 197.7 ng/L, 401.9 +/- 488.6 ng/L, 197.1 +/- 196.7 ng/L,135.0 +/- 42.8, respectively (reference range 15–65 ng/L) (Table 2).

**Table 2:** PTH values and adenoma count by anatomical location

Adenoma location	PTH, mean ± SD (ng/L)	*n*
No adenoma	94.0 ± 73.4	30
Single adenoma	136.9 ± 160.6	265
Single right	141.8 ± 197.7	143
Single left	125.9 ± 86.6	113
Single ectopic	197.1 ± 196.7	9
Multiple adenomas	372.2 ± 468.5	27
Bilateral	401.9 ± 488.6	24
Unilateral + ectopic	135.0 ± 42.8	3

*PTH *parathyroid hormone; *SD *standard deviation; *n *number of patients in each group

The Kruskal-Wallis test demonstrated significantly different PTH level depending on adenoma location (*p* < 0.001). While there was no difference in PTH level in patients with orthotopic left-sided and right-sided adenomas (*p* = 0.369) and also no difference compared to those with ectopic adenomas (*p* = 0.292), patients with bilateral adenomas had significantly higher PTH than those with other locations of adenomas (*p* < 0.001).

The total number of adenomas detected by both [^18^F]FCH PET/MRI and [^18^F]FCH PET/CT, was 322 (168 right, 143 left, and 11 ectopic), whereas surgery and histopathology confirmed 334 adenomas (178 right, 144 left, and 12 ectopic). Specifically, [^18^F]FCH PET/MRI detected a total of 266 adenomas (142 right, 116 left, and 8 ectopic) compared to 276 confirmed by reference standards (150 right, 117 left, and 9 ectopic). Meanwhile, [^18^F]FCH PET/CT detected 56 adenomas (26 right, 27 left, and 3 ectopic), with reference standards confirming 58 (28 right, 27 left, and 3 ectopic).

In the per-patient analysis of 322 patients, both [^18^F]FCH PET/MRI and [^18^F]FCH PET/CT were true positive in 283 cases, true negative in 25, false positive in 5, and false negative in 9. This resulted in an overall sensitivity of 0.969, specificity of 0.833, PPV of 0.983, NPV of 0.735, and diagnostic accuracy of 0.957.

When analysed separately, PET/MRI correctly identified 234 true positive cases and 18 true negative cases, while 4 cases were false positive and 8 were false negative. This yielded a sensitivity of 0.967, a specificity of 0.818, a PPV of 0.983, a NPV of 0.692, and a diagnostic accuracy of 0.955.

For [^18^F]FCH PET/CT alone, there were 49 true positive cases, 7 true negative cases, 1 false positive, and 1 false negative case. This resulted in a sensitivity of 0.980, a specificity of 0.875, a PPV of 0.980, a NPV of 0.875, and a diagnostic accuracy of 0.966, *p* > 0.05. (Table 3)

**Table 3:** Overall diagnostic performance of imaging methods

Parameter	[^18^F]FCH PET/MRI (95% CI)	[^18^F]FCH PET/CT (95% CI)	*p*-value
Sensitivity	0.967 (0.936–0.986)	0.980 (0.894–0.999)	> 0.05
Specificity	0.818 (0.597–0.948)	0.875 (0.474–0.997)	> 0.05
PPV	0.983 (0.960–0.993)	0.980 (0.887–0.997)	> 0.05
NPV	0.692 (0.525–0.821)	0.875 (0.497–0.980)	> 0.05
Accuracy	0.955 (0.922–0.976)	0.966 (0.881–0.996)	> 0.05

[^18^F]FCH PET/MRI ^18^F-fuorocholine positron emission tomography/magnetic resonance imaging; [^18^F]FCH PET/CT, ^18^F-fuorocholine positron emission tomography/computed tomography; *CI* confidence interval; *PPV* positive predictive value; *NPV* negative predictive value

The diagnostic performance of [^18^F]FCH PET/MRI and [^18^F]FCH PET/CT per specific setting (first- and second-line) is demonstrated in Tables 4 and 5.

**Table 4:** Diagnostic performance of [^18^F]FCH PET/MRI by setting

Parameter	Second-line (95% CI)	First-line (95% CI)	*p*-value
Sensitivity	0.979 (0.941–0.996)	0.948 (0.883–0.983)	> 0.05
Specificity	0.778 (0.400–0.972)	0.846 (0.546–0.981)	> 0.05
PPV	0.986 (0.955–0.996)	0.978 (0.927–0.994)	> 0.05
NPV	0.700 (0.419–0.883)	0.688 (0.476–0.842)	> 0.05
Accuracy	0.968 (0.926–0.989)	0.936 (0.872–0.974)	> 0.05

*CI* confidence interval; *PPV* positive predictive value; *NPV* negative predictive value

**Table 5:** Diagnostic performance of [^18^F]FCH PET/CT by setting

Parameter	Second-line (95% CI)	First-line (95% CI)	*p*-value
Sensitivity	0.973 (0.858–0.999)	1.000	> 0.05
Specificity	0.857 (0.421–0.996)	1.000	> 0.05
PPV	0.973 (0.854–0.996)	1.000	> 0.05
NPV	0.857 (0.421–0.996)	1.000	> 0.05
Accuracy	0.955 (0.845–0.994)	1.000	> 0.05

*CI* confidence interval; *PPV* positive predictive value; *NPV* negative predictive value

Out of a total of 322 patients, 296 underwent surgery, while in 26 cases surgery was withheld due to negative (*n* = 25) or inconclusive (*n* = 1) imaging results and those results were considered as false negative for the purpose of our study. In these patients, further clinical follow-up was initiated.

## Discussion

Our study compared the accuracy of [^18^F]FCH PET/MRI and [^18^F]FCH PET/CT in detecting parathyroid adenomas in patients with pHPT. Additionally, it assessed the diagnostic performance of both modalities as first-line and second-line imaging methods. Both techniques demonstrated exceptional and comparable diagnostic accuracy, exceeding 95% in both imaging settings. The overall sensitivity of both methods in our study was higher than that reported in previous studies evaluating their diagnostic performance [31, 32]. This may be attributed to the selection criteria, as we included only patients diagnosed with pHPT and excluded those with parathyroid gland hyperplasia. It is well known that[^18^F]FCH PET/MRI and [^18^F]FCH PET/CT, like all other parathyroid imaging techniques, have lower sensitivity for detecting hyperplasia [33]. While [^18^F]FCH PET/CT showed slightly higher accuracy, this difference is not considered clinically significant, given the smaller sample size in the [^18^F]FCH PET/CT group compared to the [^18^F]FCH PET/MRI group. Furthermore, our study was not able to detect a statistically significant difference between PET/CT and PET/MRI in both settings analysed. We acknowledge that, from a statistical point of view, this does not necessarily mean that both modalities are in fact equivalent [34]. This observation likely reflects the predominant influence of the PET component in hybrid imaging systems. The intense uptake of [^18^F]FCH in hyperfunctioning parathyroid tissue is the primary determinant of lesion detection, while the anatomical component (whether MRI or CT) serves as an additional localization confirmation tool rather than representing a critical factor in gland identification. However, MRI is known to provide superior anatomic localization of the parathyroid glands compared to CT [12].

Furthermore, MRI has the advantage of no radiation exposure. This is particularly important for patients undergoing multiple radiological or nuclear medicine examinations over time, as it helps minimize the cumulative radiation burden. Given an injected activity of approximately 150 MBq of [^18^F]FCH, which contributes approximately 3 mSv, PET/MRI offers a substantial reduction in radiation exposure compared to PET/CT [12]. When paired with a diagnostic CT (approximately 6 mSv), total radiation from PET/CT reaches 9 mSv – three times that of PET/MRI, which delivers only the 3 mSv from the radiopharmaceutical (67% reduction) [35]. Even when using low-dose CT protocols (approximately 1 mSv), PET/MRI still achieves a 25% reduction in total radiation exposure.

Our study provides particularly valuable data on [^18^F]FCH PET/MRI accuracy, as it is, to our knowledge, the largest study conducted to date in both first- and second-line imaging settings. Additionally, both imaging methods were evaluated by experienced, doubly-trained nuclear medicine physicians/radiologists in a tertiary institution. It is well recognized that there is typically a learning curve associated with interpreting these imaging techniques, especially in cases involving tracer uptake in cervical lymph nodes. Less frequently, mediastinal lymph nodes may also be misinterpreted as ectopic adenomas by less experienced readers [36]. Parathyroid adenomas typically show intense [^18^F]FCH uptake, while reactive lymph nodes usually demonstrate mild to moderate uptake. In contrast-enhanced CT and MRI, parathyroid adenomas are reported to show rapid, early enhancement with relatively quick washout, while lymph nodes typically show more gradual, less intense enhancement.

In terms of data analysis, all patients who tested negative on both PET imaging methods and did not undergo surgery were included in the false-negative group. This approach strengthens the validity of our findings, as the NPV could potentially be even higher with a perfect study design in which all patients undergo surgery. However, false-negative results can occur in cases of mild hypercalcemia and PTH values that fall within the upper reference range [12]. Our study confirms that patients without adenoma detection on [^18^F]FCH PET/CT(MRI) had the lowest mean PTH values compared to those with single or multiple adenomas.

Importantly, as previously shown, clear preoperative detection of pathologic parathyroid glands is more likely to lead to an increase in the number of surgeries performed in patients with pHPT [37], as observed in our study. Endocrinologists and surgeons tend to refer these patients to surgery more frequently than those with negative imaging results.

Our study revealed important differences in PTH level based on adenoma location. Patients with bilateral adenomas had significantly higher PTH levels compared to those with single adenomas. This finding aligns with previous literature suggesting that multiple gland disease typically presents with more severe biochemical profiles. Interestingly, the lack of significant PTH level difference between ectopic and orthotopic (left/right) adenomas contradicts the common assumption that ectopic adenomas would demonstrate higher PTH levels due to delayed diagnosis. This finding may be attributed to our center’s expertise in detecting ectopic adenomas using advanced cross-sectional imaging techniques such as [¹⁸F]FCH PET/MRI and [¹⁸F]FCH PET/CT, potentially leading to earlier identification before severe biochemical derangement occurs. In line with the literature, right-sided adenomas were more common than left-sided adenomas in our cohort [38].

Since at our institution, the vast majority of parathyroid lesions are histopathologically diagnosed as “adenoma” rather than “hyperplasia,” with imaging findings taken into account during diagnosis, we believe that our study well evaluates the clinical utility of [^18^F]FCH PET. During the study period, only 13 pHPT patients had been diagnosed with pure “hyperplasia”. We acknowledge that histopathological classification practices may vary across institutions. Furthermore, we recognize that the widespread use of PET imaging at our institution, particularly the improved visualization of hyperfunctioning parathyroid tissue as discrete lesions – may have introduced a bias towards classifying such findings as adenomas rather than hyperplasia. However, the distinction between adenoma and hyperplasia is, to some extent, arbitrary and carries limited clinical relevance in routine practice.

The significant age difference between patients undergoing PET/CT and those undergoing PET/MRI likely reflects a temporal evolution in pHPT diagnosis. As our study progressed - from predominantly using PET/CT in earlier years to favouring PET/MRI in later years - we observed a trend toward younger age at diagnosis. This shift likely reflects enhanced screening protocols and increased clinical awareness of pHPT among clinicians over the decade-long study period, rather than a selection bias related to imaging modality based on patient age.

Our study has several limitations. Firstly, it follows a retrospective design, although we believe this did not significantly impact our results, as supported by previous studies [31, 32]. Secondly, we did not perform lesion-based analysis; however, the patient-based analysis we conducted has true clinical relevance, as long-term outcomes depend on the accurate localization of all adenomas in patients referred for surgery. Thirdly, there was a relatively smaller number of patients in the [^18^F]FCH PET/CT group compared to the [^18^F]FCH PET/MRI, particularly in the first-line setting. This is largely due to the transition from [^18^F]FCH PET/CT to [^18^F]FCH PET/MRI, as MRI offers better anatomic resolution and reduces the radiation burden. Fourthly, as suggested by Quak et al., theoretically, more accurate data could be obtained if all patients who underwent [^18^F]FCH PET/CT(MRI) had bilateral neck exploration. However, this approach is not feasible due to obvious ethical concerns [39]. Lastly, follow-up to confirm normocalcemia and normal PTH levels after hospital discharge was not entirely possible, as patients were referred from different institutions. Moreover, different assays with varying reference values were used across laboratories during the follow-up period. However, in cases of disease recurrence, patients are typically referred for a second [^18^F]FCH PET CT/(MRI) scan, which was not observed in our patient cohort.

## Conclusions

Preoperative [^18^F]FCH PET/MRI and [^18^F]FCH PET/CT demonstrate high and comparable diagnostic accuracy in detecting parathyroid adenomas in patients with pHPT, whether used as second- or first-line imaging modalities. Both imaging techniques can be considered “top-scoring” or “one-stop-shop” methods for adenoma detection.

## Data Availability

The datasets generated during and/or analysed during the current study are available from the corresponding author on reasonable request.
